# Retraction: Emerging roles and underlying molecular mechanisms of DNAJB6 in cancer

**DOI:** 10.18632/oncotarget.28439

**Published:** 2023-07-01

**Authors:** Erhong Meng, Lalita A. Shevde, Rajeev S. Samant

**Affiliations:** ^1^Department of Pathology, University of Alabama at Birmingham, Birmingham, Alabama, USA; ^2^Comprehensive Cancer Center, University of Alabama at Birmingham, Birmingham, Alabama, USA; ^3^Beijing DOING Biomedical Technology Co. Ltd., Beijing, China

**This article has been retracted:** While re-examining this review article published in Oncotarget in 2016, it came to the authors’ attention that significant portions of the text were borrowed verbatim from other publications. Although the borrowed text was properly cited, the authors have concluded that the text in multiple places was not adequately paraphrased or appropriately quoted. In light of this, all authors have agreed to the retraction of this article from Oncotarget.

Original article: Oncotarget. 2016; 7:53984–53996. 53984-53996. https://doi.org/10.18632/oncotarget.9803

